# Pretreatment with simvastatin upregulates expression of BK-2R and CD11b in the ischemic penumbra of rats

**DOI:** 10.7555/JBR.32.20160152

**Published:** 2018-01-06

**Authors:** Jianying Zhang, Qingke Bai, Yingdong Zhang

**Affiliations:** 1. Department of Neurology, Pudong People’s Hospital, Shanghai 201299, China; 2. Department of Neurology, Nanjing First Hospital, Nanjing Medical University, Nanjing, Jiangsu 210006, China.

**Keywords:** simvastatin, cerebral ischemia/reperfusion, bradykinin B2 receptors, CD11b

## Abstract

Inhibitors of 3-hydroxy-3-methylglutaryl coenzyme A reductases, collectively known as statins, have been shown to minimize cerebral ischemic events in patients. We assessed the mechanisms of simvastatin pretreatment in preventing cerebral ischemia/reperfusion injury in rats using a model of middle cerebral artery occlusion (MCAO). Rats were pretreated with simvastatin 14 days prior to MCAO induction. At 3, 24, and 48 hours after reperfusion, bradykinin levels in the ischemic penumbra were assayed by ELISA, mRNA levels of bradykinin B2 receptors (BK-2Rs) and CD11b were measured by fluorescent quantitative real-time PCR (RT-PCR), and co-expression of microglia and BK-2Rs was determined by immunofluorescence. Simvastatin had no effect on bradykinin expression in the ischemic penumbra at any time point. However, the levels of BK-2R and CD11b mRNA in the ischemic penumbra, which were significantly decreased 3 hours after ischemia-reperfusion, were increased in simvastatin-pretreated rats. Moreover, the co-expression of BK-2Rs and microglia was confirmed by immunofluorescence analysis. These results suggest that the beneficial effects of simvastatin pretreatment before cerebral ischemia/reperfusion injury in rats may be partially due to increased expression of BK-2R and CD11b in the ischemic penumbra.

## Introduction

Statins (3-hydroxy-3-methylglutaryl coenzyme A reductase inhibitors) have neuroprotective effects against focal cerebral ischemia through lowering cholesterol^[1]^ and stabilizing plaque. Statins can also inhibit inflammatory responses^[2–3]^, exerting critical pathophysiological effects on cerebral ischemia^[4]^ and is regulated, at least in part, by the kallikrein-kinin system (KKS)^[5]^. The biological effects of KKS are mediated by B1 and B2 transmembrane receptors, coupled with different G proteins^[5]^.

Bradykinin B2 receptors (BK-2Rs) mediate most physiologic effects of kinins. These constitutive receptors are widely expressed in the brains of rats^[6–7]^ and other species, including humans^[8]^. An enhanced accumulation of neutrophils was found in the ischemic brains of BK-2R knock-out (B2R-KO) mice after MCAO compared with wild type (WT) mice^[9]^. Moreover, increased neutrophil accumulation was associated with an elevated expression of tumor necrosis factor α^[9]^. These alterations in B2R-KO mice correlated with reduced nitric oxide (NO) level, decreased Akt and glycogen synthase kinase-3β phosphorylation, and increased nicotinamide-adenine dinucleotide oxidase activity^[9]^. These results indicate that the BK-2R may provide protection against brain injury through suppressing apoptosis and inflammation induced by ischemic stroke. Functional BK receptors on microglia (including the B2 receptor subtype) play a pivotal role in the inflammatory responses of the central nervous system (CNS)^[10]^. Microglia, the motile resident immune cells of the CNS, provide immune surveillance to the brain and react to endogenous and exogenous stimuli that endanger tissue homeostasis. Ramified processes of microglia are constantly extending and retracting to explore their territories^[11–12]^. Insult to the CNS [e.g., cerebral ischemia/ reperfusion (I/R)] elicits recruitment of adjacent microglia and the expression of CD11b, leading to protective and toxic effects. Proof of concept has shown that lipocalin-2 is released by injured neurons as a distress signal, activating microglia and astrocytes to generate potentially pro-recovery phenotypes^[13]^. Ticagrelor has been shown to offer protection against ischemia-induced cerebral injury by inhibiting P2Y12-mediated microglial activation and chemotaxis^[14]^.

The concept of the ischemic penumbra was originally introduced to define the brain region that lacks sufficient blood flow to sustain cellular electrical activity yet has what is required to maintain ionic gradients^[15–16]^. Recently, this definition has been expanded to include perifocal areas that are marginally perfused, yet likely to become infarcted unless perfusion is restored or pharmacological measures are instituted to prevent additional cell death^[16]^. Therefore, penumbral tissues are considered metabolically dysfunctional, with their survival influenced by inflammatory responses associated with abnormal calcium metabolism, excitotoxic injury, enhanced free radical production, or spreading depression that progressively increases cell injury^[16–19]^.

To date, the role of simvastatin in regulating BK-2R and CD11b remains unclear. Therefore, this study was designed to assess the neuroprotective effects of simvastatin in ischemic brain injury, using a clinically relevant model of stroke in rats. We also examined whether simvastatin pretreatment modulated the in vivo expression of BK-2R and CD11b in the ischemic penumbra.

## Materials and methods

### Drug application and animals

Simvastatin (Shanghai Adamas-Beta Co., Ltd.) was suspended in 0.9% saline. Male Sprague-Dawley (SD) rats (100–120 g) were obtained from the Shanghai branch of the National Rodent Laboratory of Animal Resources of China. Rats were housed in air-conditioned rooms under a 14 hours/10 hours light/dark period, and allowed food and water *ad libitum*. The study protocol was approved by the Nanjing Medical University Experimental Animal Care and Use Committee. Every effort was made to minimize animal suffering and to reduce the number of animals used.

### Model of middle cerebral artery occlusion (MCAO)

Transient MCAO was induced as described^[20]^ with some modifications^[21]^. Briefly, rats were anesthetized by intraperitoneal (i.p.) injection of 10% chloral hydrate (0.35 mL/100 g) and subjected to MCAO for 90 minutes with an intraluminal filament. The right common carotid artery (CCA) was exposed through a lateral incision, separated from the vagus nerve, and ligated. A 4-0 nylon monofilament, with a tip rounded by heating, was introduced from the bifurcation of the internal carotid artery and advanced until resistance was felt (1.8–2.0 cm from the bifurcation). The occluder thread was removed 90 minutes after insertion. Throughout the procedure, body temperature was maintained at (37±0.5) °C with a thermostatically controlled infrared lamp. Neurological symptoms of each rat were evaluated on a 5-point grading scale, from 0 (normal) to 4 (severe systems) points^[20]^. Only rats with scores greater than 0 were used in subsequent experiments. Rats subjected to the same surgical procedure, but without the thread insertion, served as a sham-operated group.

### Experimental groups and drug application

Totally 180 male SD rats were randomized into six groups, each containing 30 rats. One group was orally administered 1 mL 0.9% saline per day (Sham). MCAO was induced in the other five groups, among which one group was treated with 0.9% saline at 1 mL/day (MCAO+ saline), one with simvastatin at 2 mg/kg/day (MCAO+ low dose), one with10 mg/kg/day (MCAO+ medium dose), one with 50 mg/kg/day (MCAO+ high dose), and one with fenofibrate at 100 mg/kg/d (MCAO+ fenofibrate). Rats were administered simvastatin or 0.9% saline 2 weeks before the onset of cerebral ischemia and weighed every other day.

Each group was further divided into three subgroups of 10 rats. At 3, 24, and 48 hours after reperfusion following a 90-minute MCAO, the rats were anesthetized with chloral hydrate (400 mg/kg, i.p.) and decapitated. Brain samples were collected with the ischemic core and penumbra dissected as described^[22]^. Cerebral penumbra cortices were immediately placed on ice, frozen in liquid nitrogen, and kept at −80 °C until analysis.

### Measurement of BK in cerebral penumbra

Each cerebral penumbra tissue sample was homogenized in a solution of 10 mmol Tris-HCl, 1 mmol EDTA, and 0.25 mol/L sucrose, pH 7.4, and centrifuged at 12,000 *g* for 10 minutes at 4 °C. BK concentrations were measured in the supernatants by ELISA (R&D) according to the manufacturer's instructions. All samples were assayed in duplicate.

### Quantitative real-time RT-PCR

Total RNA was extracted from homogenized cerebral penumbra cortices using TRIzol reagent (Invitrogen), and 1 μg of each RNA sample was reverse-transcribed to cDNA by incubation with PrimeScript^TM^ reverse transcriptase (Takara) at 37 °C for 15 minutes, followed by enzyme inactivation at 85 °C for 5 seconds. The cDNA products were stored at −20 °C until use. Quantitative SYBR Green (SYBR® Premix Ex Taq^TM^, Takara) real-time PCR was performed using equal amounts of cDNA on an ABI 7500 Real-Time PCR System (Applied Biosystems).

Primer sequences were designed using the Primer 3 software program and included primers for CD11b (sense 5′-ACATCCAGAG CACTGTCACT TATGAC-3′ and antisense 5′-TATGCCGTTC TTTGTTTCAT CAA-3′), BK-2R (sense 5′-GGTCAACTGC CCAGACACTG A-3′ and antisense 5′-AGACAGAACA CGCTGAGGAC AA-3′), and β-actin (sense 5′-GACAGGATGC AGAAGGAGAT TACT-3′ and antisense 5′-TGATCCACAT CTGCTGGAAG GT-3′). The amplification protocol consisted of an initial denaturation at 95 °C for 30 seconds, followed by 40 cycles of denaturation at 95 °C for 5 seconds, annealing and extension at 60 °C for 34 seconds. Expression was measured using the comparative Ct method and the arithmetic formula 2^−ΔΔCt^, with the levels of CD11b and BK-2R mRNAs normalized to those of β-actin mRNA in the same samples.

### Immunofluorescence

Serial coronal cryostat sections (4-μm-thick) of rat brains were obtained at the lateral striatum (optic chiasm −2.0 mm). Sections were mounted onto gelatin-coated slides and fixed in 4% paraformaldehyde at 4 °C for 15 minutes. Fixed sections were incubated overnight at 4 °C with PE-conjugated rabbit monoclonal anti-BK-2R (1/200; Bioss, Inc.) and FITC-labeled *Bandeiraea simplicifolia* /*Griffonia simplicifolia* lectin B4 (1 mg/mL, L2895, Sigma, St. Louis, MO). Control sections were incubated with nonimmune rat IgG. Each incubation was followed by washing in 0.01 mol/L PBS (pH 7.4). Sections were mounted onto the anti-fading medium Vectashield (Vector Laboratories, Burlingame, CA), and viewed under a fluorescence microscope.

### Statistical analysis

All statistical analyses were performed using SPSS 13.0 software. Results were expressed as means±standard deviations (SDs) and examined for homogeneity of variance. Comparisons between two groups were made by Student’s *t*-tests and among multiple groups were by one-way analysis of variance (ANOVA). A *P*-value <0.05 was considered statistically significant.

## Results

### Expression of BK in cerebral penumbra

**Fig.1:**
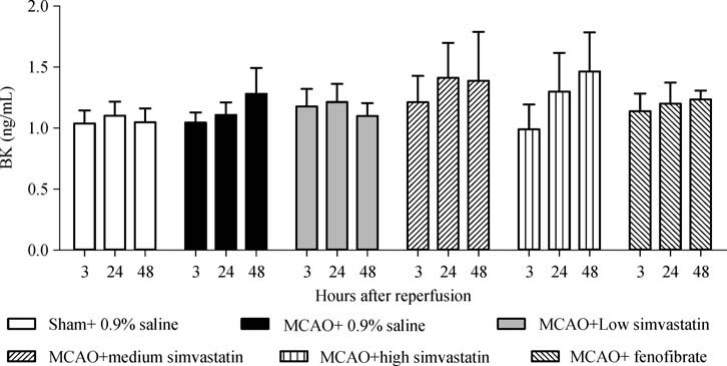
Expression of bradykinin (BK) in cerebral penumbra.

ELISA in the rat cerebral penumbra tissues at 3, 24, and 48 hours after cerebral I/R showed that cerebral infarction did not significantly alter BK expression compared with the sham-operated rats (*P*>0.05). Moreover, pretreatment of rats with 2, 10, or 50 mg/kg/day of simvastatin or 100 mg/kg/day of fenofibrate did not alter BK expression compared with the group pretreated with 0.9% saline at any time point after cerebral I/R (*P*>0.05, ***Fig. 1***).

### Expression of BK-2R and CD11b mRNA

**Fig.2:**
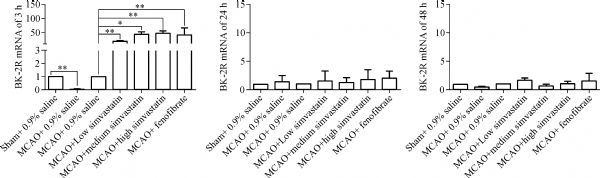
Expression of BK-2R mRNA in cerebral penumbra tissues.

**Fig.3:**
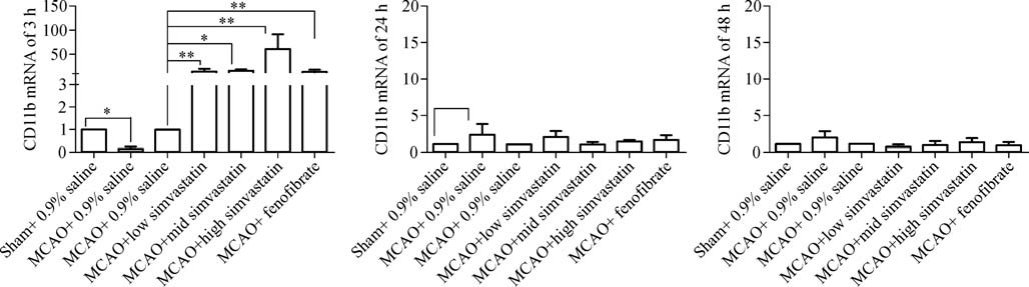
Expression of CD11b mRNA in cerebral penumbra tissues.

The levels of expression of BK-2R and CD11b mRNAs in the cerebral ischemic penumbra were analyzed by quantitative real-time RT-PCR. Both BK-2R and CD11 mRNA levels were lower in MCAO-treated, saline-pretreated rats 3 hours after reperfusion than in sham-operated, saline pretreated rats (BK-2R 3 hours: Sham, 1.00±0.00; MCAO, 0.03±0.01; CD11 3 hours: Sham, 1.00±0.00; MCAO, 0.15±0.10; *P*<0.01), but were similar after 24 and 48 hours (BK-2R 24 hours: Sham, 1.00±0.00; MCAO, 1.44±1.03; BK-2R 48 hours: Sham, 1.00±0.00; MCAO, 0.46±0.21; CD11 24 hours: Sham, 1.00±0.00; MCAO, 2.30±1.50; CD11 48 hours: Sham, 1.00±0.00; MCAO, 1.89±0.85; *P*>0.05). Pretreatment of MCAO-induced rats with three concentrations of simvastatin or with fenofibrate increased BK-2R and CD11b mRNA levels 3 hours after cerebral I/R compared with rats pretreated with 0.9% saline (BK-2R 3 hours: MCAO, 1.00±0.00; MCAO+ low simvastatin, 19.15±1.48; MCAO+ medium simvastatin, 44.47±7.05; MCAO+ high simvastatin, 47.48±7.85; MCAO+ fenofibrate, 42.20±23.90; CD11 3 hours: MCAO, 1.00±0.00; MCAO+ low simvastatin, 7.71±5.82; MCAO+ medium simvastatin, 8.71±2.49; MCAO+ high simvastatin, 57.11±31.31; MCAO+ fenofibrate, 7.23±4.47). However, BK-2R and CD11b mRNA levels were similar in these five groups 24 and 48 hours after cerebral I/R (BK-2R 24 hours: MCAO, 1.00±0.00; MCAO+ low simvastatin, 1.55±1.74; MCAO+ medium simvastatin, 1.24±0.90; MCAO+ high simvastatin, 1.78±1.73; MCAO+ fenofibrate, 2.01±1.26; BK-2R 48 hours: MCAO, 1.00±0.00; MCAO+ low simvastatin, 1.68±0.42; MCAO+ medium simvastatin, 0.07±0.27; MCAO+ high simvastatin, 1.09±0.40; MCAO+ fenofibrate, 1.57±1.29; CD11 24 hours: MCAO, 1.00±0.00; MCAO+ low simvastatin, 2.01±0.83; MCAO+ medium simvastatin, 0.99±0.33; MCAO+ high simvastatin, 1.42±0.16; MCAO+ fenofibrate, 1.67±0.62; CD11 48 hours: MCAO, 1.00±0.00; MCAO+ low simvastatin, 0.63±0.31; MCAO+ medium simvastatin, 0.88±0.54; MCAO+ high simvastatin, 1.22±0.58; MCAO+ fenofibrate, 0.79±0.45; *P*>0.05, ***Fig. 2*** and ***Fig. 3***).

### Immunofluorescence analysis of BK-2R and CD11b expression levels in cerebral penumbra tissues

**Fig.4:**
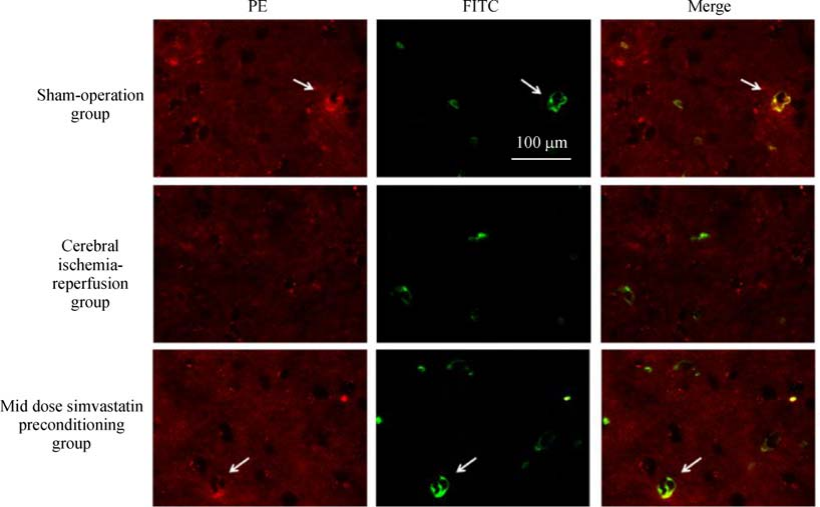
Immunofluorescence analysis of BK-2R and CD11b expression levels in cerebral penumbra tissues.

The expression of BK-2R in rat microglia was confirmed by immunofluorescence using a PE-conjugated rabbit monoclonal anti-BK-2R antibody and the microglial marker, FITC-conjugated *B. simplicifolia*/*G. simplicifolia* lectin B4. As reported in other cells^[23]^, BK-2R expression was distributed heterogeneously, with patches of punctate labeling separated by areas with apparent little labeling (***Fig. 4***). Simultaneous viewing of the staining patterns revealed a close relationship between expression of the microglial marker protein and BK-2R, indicating that many, but not all, microglial cells expressed BK-2R (***Fig. 4*** and ***Table 1***).

**Tab.1:** Number of BK-2R and/or CD11-positive cells (mean±SD)

Groups	BK-2R-positive	CD11-positive	BK-2R- and CD11-positive
Sham	48.13±11.70	6.50±2.27	0.63±0.74
MCAO	27.63±6.30	3.13±0.64*	0.25±0.46
MCAO+ medium simvastatin	47.50±14.85^#^	7.13±3.00^#^	1.75±1.28^#^

*P<0.05 compared with Sham; #*P*<0.05 compared with MCAO.

## Discussion

Previous studies have reported that the KKS displays both anti-inflammatory and neuroprotective effects in the CNS^[24]^, suggesting that the KKS may have beneficial or harmful effects after focal cerebral I/R in rats. B1and B2 receptors are potential therapeutic targets in several inflammatory related pathophysiological events, such as pain, sepsis, allergic asthma, rhinitis, and edema, as well as diabetes and cancer^[25]^. Following experimental stroke, BK is produced in the brain, kinin B2 receptors are upregulated in dying cells, and B2 receptors are involved in cell death and brain edema formation^[26–27]^. However, whether BK-2R has neuroprotective effects in strokes remains unclear. BK-2R has been reported to promote survival and protect the brain from injury by suppressing apoptosis and inflammation induced by ischemic stroke^[28]^. Similar promoting and protective mechanisms have also been found in kinin B2 receptor^[9]^. In contrast, BK-2R has been found to mediate CNS cell death and brain edema formation after experimental stroke^[27]^, and a bradykinin B2 receptor antagonist has been found to reduce ischemic brain injury in a murine model^[29]^.

BK-2Rs have been reported to have both beneficial and harmful effects on the pathogenesis of nervous system diseases^[9, 25]^, while further research is still needed to assess their precise effects on brain injury following cerebral ischemia. Bradykinin (BK) receptors are expressed in a wide variety of cell types, including vascular endothelial cells, neurons, smooth muscle cells, certain leukocytes, and microglial cells. We observed that simvastatin pretreatment upregulated BK-2R in MCAO rats, suggesting that BK-2R has neuroprotective effects, at least in our model. Microglia represent a highly responsive population of cells that can regulate adaptive immunity and participate in homeostatic anti-inflammatory mechanisms^[30]^. Treatment of rats with gliotoxin fluorocitrate showed that disabling the glia reproduced many features of ischemic penumbra^[31]^. For example, neurons became highly vulnerable in the absence of normal glia, suggesting that early glial dysfunction was associated with neuronal loss and indicating the role of glial cells in neuronal function and survival^[31]^. Neuron-to-microglia communication may play a key role in shaping the quiescent and reactive states of microglia. The presence of numerous receptors for CNS signaling molecules and neurotrophic factors on microglia suggests that these cells not only monitor, but are also controlled by the neurochemical environment^[30]^. Ipocalin-2 is released by injured neurons as a distress signal that activates microglia and astrocytes, generating potentially prorecovery phenotypes^[13]^.

In this study, simvastatin pretreatment of MCAO-treated rats upregulated the expression of CD11b, a marker of microglia. BK-2R was expressed on microglia in vivo. However, we could not rule out the possibility that the enhanced CD11b expression might be caused by the increase in the number of CD11b expressing cells. Although the functions of BK-2R in microglia are not known, they may play a key role in cell migration in response to pathophysiological conditions resulting from stroke. ELISA results showed that pretreatment with simvastatin had no effect on BK expression in the ischemic penumbra of rats at any time point. Fluorescent quantitative RT-PCT analysis indicated that the expression levels of BK-2R and CD11b mRNAs decreased 3 hours after I/R, but not at later times, compared with sham-operated animals. Simvastatin pretreatment increased the expression levels of BK-2R and CD11b mRNAs 3 hours after I/R, but not at later times, compared with animals treated with MCAO and 0.9% saline. Immunofluorescence analysis of the ischemia penumbra confirmed the co-expression of BK-2R and microglia.

Treatment with statins has been found to reduce the risk of ischemic stroke in patients at increased risk of vascular disease. Statins have been shown to possess neuroprotective properties in acute cerebral ischemia. Despite our finding that simvastatin pretreatment upregulated the expression of the functionally protective molecules BK-2Rs and CD11b, the mechanisms underlying these effects remain largely unknown.
